# Seroprevalence and risk factors of brucellosis in abattoir workers at Debre Zeit and Modjo export abattoir, Central Ethiopia

**DOI:** 10.1186/s12879-017-2208-0

**Published:** 2017-01-26

**Authors:** Amanuel Tsegay, Getachew Tuli, Tesfu Kassa, Nigatu Kebede

**Affiliations:** 1 Akillu Lemma Institute of Pathobiology (ALIPB), Addis Ababa University (AAU), P. O. Box: 150480, Addis Ababa, Ethiopia; 2 National Animal Health Diagnostic and Investigation Center (NAHDIC), Sebeta, Ethiopia

**Keywords:** Abattoirs workers, Brucellosis, CFT, RBPT, Seroprevalence, Ethiopia

## Abstract

**Background:**

Brucellosis is one of the major zoonoses globally with great veterinary and public health importance, particularly in developing countries where people are having frequent contact with livestock and animal products. This cross sectional study was carried out from November 2013 to May 2014 to determine the seroprevalence and assess the potential risk factors of brucellosis in abattoir workers of five export abattoirs at Debre Ziet and Modjo, Central Ethiopia.

**Methods:**

Serology and structured questionnaire were the methods used. In this study, 156 abattoir workers participated in the questionnaire survey and among them, 149 agreed for blood sample collection. Rose Bengal Plate Test and Complement Fixation Test were conducted using sera samples at serology laboratory of the National Animal Health Diagnostic and Investigation Center. Data collection sheets were used to gather information on possible risk factors believed to influence the spread of *Brucella* infection in abattoir workers such as sex, age, marital status, duration on job, types of work, educational level, etc. and further information obtained include knowledge of brucellosis and other zoonotic diseases infection, symptoms of the disease, milk and meat consumption habits and work related risk factors. Chi-square and Fisher’s exact tests were used for data analysis.

**Results:**

The overall seroprevalence of brucellosis in abattoir workers was found to be 4.7 and 1.3% using Rose Bengal plate test and Compliment fixation test, respectively. Based on the questionnaire survey, 66 (44.2%) and 85 (53.21%) of abattoir workers were aware of brucellosis and other zoonotic diseases, and 29 (18.6%) and 21 (13.5%) were using gloves and cover their mouth while slaughtering, respectively.

**Conclusion:**

Brucellosis in abattoir workers could be prevented by using protective closing and measures. Concerned body should educate occupationally exposed groups and the general public regarding e prevention and control of brucellosis and other zoonotic diseases.

## Background

Nowadays, our world has been threatened by numerous emerging and re-emerging pathogenic diseases; these diseases are seriously affecting the well being of human, animal health, and animal production [1]. Most of them are zoonotic diseases and have great veterinary and public health impact, particularly in developing countries where people are having daily frequent contact with livestock and animal products. Brucellosis is one of them, and it causes significant economic losses due to abortion, lower milk production and reduced fertility in livestock and serious health problem in human beings [2]. It is one of the most widespread and the second most important global zoonoses after rabies [3–5]. Brucellosis is endemic in many parts of the world including Latin America, the Middle East, western Asia, part of Mediterranean regions and Africa [6]. Ever increasing international trade of animal and animal products, movement of people, and urbanization significantly contributed for the transmission of the diseases between animals and humans [7].

Brucellosis is more prevalent in developing countries and considered to be a serious health problem due to lack of effective public health measures, domestic animal health programs, and appropriate diagnostic facilities. Furthermore, the situation is also worsened by the resemblance of the disease with other diseases leading to misdiagnosis and under-reporting [8].

Brucellosis is an infectious bacterial disease caused by members of the genus *Brucella* and characterized as small, facultative, gram negative, non-motile, non-spore forming and rod shaped (coccobacilli) bacteria [9, 10]. Human beings are susceptible to infection with *B. abortus, B. melitensis, B. suis biovars* 1*–*4*,* and rarely *B. canis;* however, *B. melitensis* causes serious infection and is responsible for the most of worldwide morbidity [11]. Brucellosis in humans is a systemic disease characterized by acute or insidious onset of continued, intermittent, undulent or irregular fever of variable duration, headache, profuse sweating, chills, weakness, generalized aching, and joint pain [12].

From public health point of view, brucellosis is considered to be an occupational disease that mainly affects abattoir workers, farm laborer, animal keepers, butchers, veterinarians and laboratory workers [13]. However, abattoir workers are more prone to acquire brucellosis than other occupations, because they are over exposed to carcasses, viscera, and organs of infected animals [8]. Mukhtar and Kokab, [8] and Karimi et al. [14] reported a prevalence of brucellosis among slaughter house workers, 21.7% in Pakistan and 35.7% in Saudi Arabia, respectively.

In Ethiopia, a number of zoonotic diseases are known to have public health and economic importance both in humans and animals. The present study has particularly given emphasise to brucellosis because of the high morbidity and its significance to the public health and the economy of the country; brucellosis is of high national priority. Like other developing countries, brucellosis has not been brought under control in Ethiopia in livestock, which might be due to mismanagement on animal quarantine, trans-boundary animal movement, a lack of eradication and vaccination program, and that of awareness of the disease among pastoralist, farmers and general public [15]. Very few studies were carried out to determine seroprevalence of brucellosis in humans, particularly occupational exposed group in Ethiopia. Some of the previous studies indicated that there is a prevalence of brucellosis in pastoralist, farmers and occupational exposed groups. Tolosa et al. [16], Kassahun et al. [17], Hailemelekot et al. [18], Kassahun et al. [19] and Haileselassie et al. [20] reported prevalence of human brucellosis of 2.4, 5.3, 3.8, 4.8 and 1.2% in different parts of the country, respectively. However, the prevalence of human brucellosis in Ethiopia is not well documented. In addition, with the exception of few studies, awareness level of brucellosis and its public health significance among occupational exposed group is almost unknown. Hence, this study was aimed at estimating the seroprevalence of brucellosis in abattoir workers and assesses the factors predisposing abattoir workers to brucellosis in selected export abattoirs at Debre Zeit and Modjo, Central Ethiopia.

## Methods

### Study areas

There are seven functional export abattoirs found in Ethiopia, and five of them are located in the cities of Debre Zeit and Modjo, Central Ethiopia. This investigative work has been done in five export abattoirs located at Debre Zeit and Modjo due to their proximity to Addis Ababa and Sebeta where National Animal Health Diagnostic and Investigation Center is located. Debre Zeit is approximately 47 km south east of Addis Ababa, and found at 9° N and 40°E with an altitude of 1880 m above sea level. It has an annual rainfall of 1151.6 mm, and its mean annual minimum and maximum temperature is 8.5° C and 30.7 °C, respectively, and the mean humidity is 61.3%. Modjo is about 77 km south east of Addis Ababa located at 8°35′N and 39° 10′ E at an altitude of 1777 masl. The average maximum and minimum temperature is 28 and 18° C [21].

### Study design

A cross sectional study was employed from November 2013 to May 2014 to determine the seroprevalence of brucellosis and assess the associated risk factors to *Brucella* seropositivity and to determine the awareness level of brucellosis among export abattoirs workers.

### Sample size and study subjects

The sample size was calculated using the method recommended by Thrusfield, [22]. The sample size for abattoir workers were determined based on the recent seroprevalence studies of brucellosis in Ethiopia. According to Negash et al. [1] and Kassahun et al. [19], a prevalence of 4.8% was considered to determine sample size. Accordingly, the minimum samples 70 with 95% confidence interval and 5% margin of error. However, to increase precision level the numbers of abattoirs workers to be examined were increased to 156.

The formula for calculating sample size:-

$$ n={Z_{\frac{a}{2}}}^2\frac{P\left(1- P\right)}{d^2} $$

Where;

n = the minimum sample size required for very large population

Z = the critical value for a given confidence interval

P = expected proportion of the event to be studied

d = margin of error (the margin of error is 0.05)

Study subjects were assigned proportionally to each abattoir, based on the number of workers that particular abattoir employed. After getting the desired sample size for each abattoir, stratified sampling techniques were employed to determine sampling population in each category. After getting the job description of workers of export abattoirs, each worker was allocated to his/her specific strata based on his/her type of job into five classes such as: veterinarian, slaughterer, loader, cleaner and animal keeper. Then sample was drawn from each strata proportionally using systematic random sampling to get the final sample size.

Abattoir workers of the five export abattoirs who have direct contact with live, carcasses, organs and viscera of animals were study population. One hundred fifty six employees were selected as a study subject and interviewed by the principal investigator; however, blood samples were taken only from 149 respondents who were voluntary to give blood samples.

### Eligibility criteria

#### Inclusion criteria

Employees of the five export abattoirs who have direct contact with live and carcasses of ruminants; workers who can understand questionnaire and sign the informed consent and the age above 18 year old.

#### Exclusion criteria

Temporary workers; workers not voluntary to participate in the study and individuals in the administrative and managerial posts.

### Questionnaire survey

A structured questionnaire was used to gather information on socio-demographic characters of the respondents such as: sex, age, marital status, duration on job, types of work and etc.; and further information obtained include level of awareness of brucellosis and other zoonotic diseases infection, symptoms of the disease, milk and meat consumption habits and work related risk factors. The questionnaire was translated to local language and administered by the principal investigator in order to minimize bias by the interviewer.

### Blood sample collection

Approximately 3–5 ml of venous blood was drawn from each respondents using plain vacutainer tube (BD vacutate, UK), syringe and needles using aseptic techniques. Each vacutainer tube was marked with a permanent marker recording the case number of the respective study subject and the name of the abattoirs. The blood samples were placed at an inclined position and allowed to clot for 1–2 h at room temperature in order to get clear serum and to minimize hemolysis of blood. Then stored overnight horizontally at 4 °C, the serum was separated from the clot by centrifugation at 2500 rpm for 10 min at room temperature. Finally, the serum was transferred to a labeled vial and stored in deep freezer (−20 °C) until further test was done. Blood samples were taken by qualified medical personnel.

### Serological tests

Two types of serological tests were employed as a screening and confirmatory test for the detection of *Brucella* antibody in human serum, the Rose Bengal plate Test (RBPT) and Compliment Fixation Test (CFT), respectively. Both serological tests were done at the National Animal Health Dagnostic and Investigation Center (NAHDIC).

### Rose Bengal plate tests

RBPT was used as a screening test for the serum samples collected for the presence of *Brucella* agglutinins. The test was conducted as per recommended procedure [23, 24]. Equal volumes of test serum and *Brucella* abortus antigen Strain 99 (LD, UK) (30 μl) were placed alongside in a plate and then mixed thoroughly with tooth pick then rocked for 4 min with hands. Then the results were interpreted according to the presence and degree of agglutination. Samples with no agglutination (0) were recorded as negative while those with +, ++, and +++ were recorded as positive.

### Complement fixation tests

All sera tested positive using RBPT were further confirmed using CFT. The complement system consists of series of proteins that, if triggered by an antigen antibody complex, react in a sequential manner to cause cell lysis. The test has two steps. The first step is antigen, test serum and complement are mixed and incubated. The second step is an indicator system which consists of sheep red blood cells (SRBC) and amboceptor that sensitizes red blood cells to the action of the complements. If the test serum contains antibodies to *Brucella,* an antigen-antibody complex is formed; the complement is used up and no lysis of SRBC occurs. If the test serum does not contain *Brucella* antibodies (negative reaction), the complement will not be fixed and lysis of SRBC would occur [23].

### Data collection and data analysis techniques

Results from serological tests, questionnaires and data collection sheets were coded and stored in EpiData version 3.1, and then exported to STATA (Stata Corp, Texas, USA) version 11.0 for data analysis. Positive samples confirmed using CFT were considered as true seropositive and taken for further data analyses. The test agreement between RBPT and CFT was analyzed using Kappa statistics. Categorical data were expressed in percentage, and seroprevalence was calculated by dividing the number of positive sera samples by the total samples examined. Odds ratio, 95% confidence interval, and Chi-square and Fisher’s exact tests were computed to see the degree of association of the risk factors with *Brucella* seropositivity. For all analysis, a *p* value < 0.05 was taken as significant. In the present study five export abattoirs have participated, namely, Hashim, Elfora, Luna, Organic and Modjo export abattoirs. In order to insure confidentiality of the participating abattoirs, code numbers were used.

## Results

Of the 149 human sera samples screened with RBPT, 7 (4.70%) were found positive. Samples screened positive for RBP further confirmed with CFT where 2 (1.34%) were confirmed positive. Relatively high *Brucella* seropositivity was observed in female respondents than in male, and within the age group of 21–30 years old than other age groups. On residential background, high seroprevalence was recorded in workers with rural background than urban (Table 1). Only Moderate agreement [Kappa = 0.4326] was observed between RBPT and CFT for the detection of *Brucella* antibody in abattoir workers. High *Brucella* seropositivity was found among respondents who work from 6 to 10 years on job, primary school background and cleaner (Table 2).

**Table 1:** Demographic characterstics and seroprevalence of brucellosis in abattoir workers of Debre Zeit and Modjo export abattoirs (*N* = 149)

Category	*N*	RBPT positive *N* (%)	CFT positive *N* (%)	95% CI for CFT	Fisher’s exact *P*-value
Sex					
Male	122	5 (4.10)	1 (0.82)	0.02–4.48	0.331
Female	27	2 (7.41)	1 (3.70)	0.09–18.97	
Age (in years)					
< =20	19	1 (5.26)	-		
21–30	75	5 (6.49)	2 (2.6)	0.32–9.07	1
31–40	*35*	-	-		
41–50	9	1 (11.11)	-		
>50	9	-	-		
Marital status					
Single	57	5 (8.77)	1 (1.75)	0.04–9.39	1
Married	89	2 (2.25)	1 (1.12)	0.03–6.10	
Divorced/widowed	3	-	-		
Background					
Urban	120	5 (4.17)	1 (0.83)	0.02–4.56	0.352
Rural	29	2 (6.90)	1 (3.45)	0.09–17.46	
Total	149	7 (4.70)	2 (1.34)	0.16–4.76

**Table 2:** Seroprevalence of brucellosis among Debre Zeit and Modjo abattoirs workers by years on job, level of education, types of job and name of abattoir (*N* = 149)

Category	*N*	RBPT positive *N* (%)	CFT positive no (%)	95% CI for CFT	Fisher’s exact *P*-value
Years on job					
<1	19	1 (5.26)	-		
1–5	82	3 (3.66)	-		
6–10	33	3 (9.09)	2 (6.06)	0.74–20.23	0.173
11–15	11	-	-		
>15	4	-	-		
Level of education					
Illiterate	13	1 (7.69)	-		
Primary	54	3 (5.56)	2 (3.70)	0.45–12.75	0.546
Secondary	60	3 (5.00)	-		
Post secondary	22	-	-		
Types of job					
Animal keepers	23	-	-		
Slaughterers	60	1 (1.67)	-		
Loaders	31	2 (6.45)	1 (3.23)	0.08–16.70	0.355
Veterinarian	12	-	-		
Cleaners	23	4 (17.39)	1 (4.35)	0.11–21.95	
Name of abattoirs					
Abattoir 1	30	-	-		
Abattoir 2	27	1 (3.70)	1 (3.70)	0.09–18.97	
Abattoir 3	30	1 (3.33)	1 (3.33)	0.08–17.21	0.342
Abattoir 4	31	5 (16.13)	-		
Abattoir 5	31	-	-		

Questionnaire survey was conducted on 156 abattoir workers about their knowledge on brucellosis and other zoonotic diseases. Of these, 125 (80.1%) were men whilst 31 (19.9%) were women. The overall awareness level of abattoir workers toward brucellosis and other zoonotic diseases was 69 (44.2%) and 83 (53.2%), respectively (Table 3). However, there was no statistically significant difference in the knowledge about brucellosis and other zoonotic diseases among gender, age, marital status, residential background and duration on job (*p* > 0.05).

**Table 3:** Awareness level of abattoir workers on brucellosis and other zoonotic diseases in Debre Zeit and Modjo export abattoirs (*N* = 156)

Category	*N*	Brucellosis (yes) *N* (%)	*χ* ^2^	*P*-Value	Zoonosis (yes) *N* (%)	*χ* ^2^	*P*-Value
Sex							
Male	125	55 (44.00)	0.013	0.907	68 (54.40)		
Female	31	14 (54.84)			15 (48.39)	0.361	0.548
Age (in years)							
< =20	21	3 (14.29) ^a1^			7 (33.33)		
21–30	80	35 (43.75) ^a^	12.075	0.017	44 (55.00)		
31–40	*37*	19 (51.35) ^b^			19 (51.35)	6.323	0.176
41–50	9	6 (66.67) ^b^			6 (66.67)		
>50	9	6 (66.67) ^b^			7 (77.78)		
Marital Status							
Single	60	22 (36.67)			32 (53.33)		
Married	92	43 (46.74)	6.670	0.036	48 (52.17)	0.803	0.669
Divorced/Widowed	4	4 (100)			3 (75.00)		
Back Ground							
Urban	125	58 (46.40)			68 (54.40)		
Rural	31	11 (35.48)	1.200	0.273	15 (48.39)	0.361	0.548
Years On Job							
<1	19	5 (26.32)			9 (47.37)		
1–5	86	35 (40.70)	7.626	0.106	45 (52.33)	1.193	0.879
6–10	35	18 (51.43)			19 (54.29)		
11–15	12	8 (66.67)			7 (58.33)		
>16	4	3 (75.00)			3 (75.00)		
Total	156	69 (44.23)			83 (53.21)		

^1^Within a variable, values in the same column with different letters differ significantly

Knowledge of brucellosis and other zoonotic diseases of post secondary workers was 15 (68.2%) and 19 (86.5%), respectively, while that of veterinarian was 12 (100%). Statistically significant difference was observed in the knowledge about brucellosis and other zoonotic diseases of those post secondary school background with the other level of education, respectively (*p* = 0.010 and *p* = 0.001). Similarly, statistically significant difference was observed in the knowledge about the diseases by that of the veterinarians than other worker classes (*p* = 0.001) and (*p* = 0.009) (Table 4).

**Table 4:** Knowledge of Debre Zeit and Modjo abattoirs workers about brucellosis and other zoonotic diseases by year on job, level of education, types of job, and abattoirs (*N* = 156)

Category	*N*	Brucellosis (yes) *N* (%)	*χ* ^2^	*P*-Value	Zoonosis (yes) *N* (%)	*χ* ^2^	*P*-Value
Level of education							
Illiterate	13	6 (46.15)^a^			5 (38.46)^a^		
Primary	56	6 (28.57) ^a^	11.362	0.010	21 (37.50)^a^	17.120	0.001
Secondary	65	32 (49.23)^b^			38 (58.46)^b^		
Post Secondary	22	15 (68.18)^b^			19 (86.36)^b^		
Types of Job							
Animal Keepers	23	8 (34.78)^a^			12 (52.17)^a^		
Slaughterers	64	30 (46.88)^a^			35 (54.69)^a^		
Loaders	33	11 (33.33)^a^	18.888	0.001	4 (42.42)^a^	13.444	0.009
Veterinarian	12	12 (100)^b^			12 (100)^b^		
Cleaners	24	8 (33.33)^a^			10 (41.67)^a^		
Name of abattoirs							
Abattoir 1	32	14 (43.75)			15 (46.88)		
Abattoir 2	27	15 (55.56)			14 (51.85)		
Abattoir 3	31	9 (29.03)	4.742	0.315	20 (64.52)	2.742	0.602
Abattoir 4	34	15 (44.12)			21 (61.13)		
Abattoir 5	32	16 (50)			17 (53.13)		
Total	156	69 (44.23)			83 (53.21)		

^1^Within a variable, values in the same column with different letters differ significantly

Regarding questions on predisposing factors for brucellosis, out of 156 respondents 29 (18.6%) and 21 (13.4%) replied using gloves and cover their mouth during slaughtering and eviscerating process, respectively. In addition, 109 (69.9%), 107 (68.6%) and 76 (48.7%) of the respondents consumed raw dairy products and meat, and accidentally cut during slaughtering and eviscerating, respectively (Table 5).

**Table 5:** Perceived predisposing factors Debre Zeit and Modjo abattoirs workers to *Brucella* seropositivity (*N* = 156)

Risk factors	N interviewed	Yes *N* (%)	No *N* (%)
Use of gloves	156	29 (18.59)	127 (81.41)
Mouth cover	156	21 (13.46)	135 (86.54)
Hand wash	156	152 (99.36)	1 (0.64)
Accidental cut	156	76 (48.72)	80 (51.28)
Treatment of sore	76	64 (84.21)	12 (15.79)
Assist parturition	156	14 (8.97)	142 (91.03)
Consumption of raw meat	156	109 (69.87)	47 (30.13)
Consumption of raw Milk and milk products	156	107 (68.59	49 (31.41)
Own livestock	156	29 (18.59)	127 (81.41)

## Discussion

The present investigation found an indication for prevalence of brucellosis and the relatively low level of awareness of export abattoir workers about brucellosis and other zoonotic diseases. Overall, 149 abattoir workers sera samples were examined for the presence of brucellosis. Of the total examined, prevalence of brucellosis using RBPT and CFT was found to be 4.7 and 1.3%, respectively. Observed differences between the two tests might be due to cross reaction of *Brucella* with other bacteria which share similar epitopes. In Ethiopia, very few studies have been conducted to determine prevalence of brucellosis in humans particularly in abattoir workers. The few similar studies in the literature, particularly the work done in association with occupational groups as in the present study, reported prevalence of 16 (4.8%) and 3 (1.2%) brucellosis, in Addis Ababa and Western Tigray slaughter houses, respectively [19, 20]. Compared to the present study, however, the relatively higher seroprevalence of brucellosis observed by Kassahun et al. [19] might be attributed to the the large sample size involved and/or the different confirmatory tests used by the two studies, CFT versus 2–mercaptoethanol test (MET). Rather, very high *Brucella* infection was reported in abattoir workers in other countries, Agasthya et al. [25] and Mukhtar and Kokab [8] reported prevalence of 97 (19.69%) and 78 (21.7%) among slaughter house workers in India and Pakistan, respectively. This might be correlated to low prevalence of small ruminant brucellosis reported in these abattoirs [26]. Since seropositivity of brucellosis in human is largely affected by the presence of the disease among domestic animals [11].

Concerning knowledge of brucellosis and other zoonotic diseases, questionnaire based data were collected from the selected five export abattoir workers.. The overall awareness level of brucellosis and other zoonotic diseases among export abattoir workers were found to be relatively low. In addition, most of the workers do not wear protective clothing while slaughtering and eviscerating animals and considerable portion of abattoir workers consumed raw meat and dairy products. Similar finding was also reported by Tuli, [27] among export and municipal abattoir workers in Debre Zeit. This might be attributed to their educational status, since most of the workers were illiterate or primary education background. Additionally, it could also be due to a lack of awareness creation program about zoonotic diseases among abattoir workers.

Data analysis has been done using Chi-square test and *p*-value to see if there were associations between different categories of workers and awareness level of brucellosis and other zoonotic diseases. Statistically significant differences were observed in the level of knowledge of brucellosis and other zoonotic diseases of workers with post secondary school background and veterinarian with their respective classes. The high level of awareness of brucellosis and other zoonotic diseases among veterinarian and those post secondary school background were as expected. Because veterinarians and workers with post secondary education background have considerable theoretical knowledge about the diseases during their professional training and also have good amount of practical knowledge through their experiences. Similarly, statistically significant difference was also observed among different age groups of the respondents toward awareness of brucellosis. This could be due to the age of the workers whereby older people be more aware of the disease than the young ones, through their lifelong experiences. On the other hand, statistically significance difference was not observed among other categories of abattoir workers with regard to knowledge of brucellosis and other zoonotic diseases, despite considerable variation in their knowledge about the diseases.

Generally, a lack of sufficient knowledge of brucellosis and other zoonotic diseases, unprotective working conditions, regular exposure from aerosol and contact through cuts and abrasion to infected materials such as carcasses, viscera, organs, blood and urine are considered as fertile grounds for exposure and transmission of the diseases to humans. In this regard, very little has been done by way of awareness creation of brucellosis and other zoonotic diseases, and as also the consumption of raw milk and meat products would lead export abattoir workers at high risk of exposure to *Brucellosis* and other zoonotic diseases [28].

## Conclusion

This study showed the presence of seropositive abattoir workers which indicates that there are sources/foci of infection which may spread to general public. Meanwhile, the present study also revealed that working in abattoir is a risk factor to *Brucella* infection. Workers in these abattoirs also do not take proper preventive measures while slaughtering and eviscerating animals and have relatively low awareness level of the diseases. This could create the risk of infection and exposure to brucellosis and other zoonotic diseases. Therefore, awareness about brucellosis and other zoonotic diseases should be created and promoted to abattoir workers. Finally, further studies should be carried out in other parts of the country not only in abattoirs, but also in general population associated with livestock to estimate the status of brucellosis and to assess also the level of awareness of the disease among occupationally exposed groups in the whole country.

## Abbreviations

CFT: Compliment fixation test
d: Margin of error
n: The minimum sample size required for very large population
NAHDIC: National Animal Health Dagnostic and Investigation Center
P: Expected proportion of the event to be studied
RBPT: Rose Bengal plate test
Z: The critical value for a given confidence interval
